# Psychosocial and productivity impact of caring for a child with peanut allergy

**DOI:** 10.1186/s13223-020-00477-3

**Published:** 2020-09-25

**Authors:** Sarah Acaster, Katy Gallop, Jane de Vries, Anne Marciniak, Robert Ryan, Andrea Vereda, Rebecca Knibb

**Affiliations:** 1 Acaster Lloyd Consulting Ltd, 16 Woburn Pl, Bloomsbury, London, WC1H 0BS UK; 2 Independent Consultant to Aimmune Therapeutics, London, UK; 3 Aimmune Therapeutics, 10 Eastbourne Terrace, London, W2 6LG UK; 4 Department of Psychology, School of Life and Health Sciences, Aston University, Aston Triangle, Birmingham, B4 7ET UK

**Keywords:** Peanut allergy, Caregiver, Parental burden, Health-related quality of life, Productivity, Psychosocial burden

## Abstract

**Background:**

Limited previous research has assessed the psychosocial burden and productivity impact of caring for a child with peanut allergy and factors associated with burden. The objective of this research was to explore caregiver burden in terms of psychosocial and productivity impact of caring for a child with peanut allergy, the influence of caregiver and child gender on caregiver burden, and factors predicting caregiver burden in peanut allergy.

**Methods:**

A cross-sectional survey of caregivers of children with peanut allergy was conducted in the United Kingdom, and included sociodemographic and clinical questions, EQ-5D, Hospital Anxiety and Depression Scale, Food Allergy Quality of Life-Parental Burden, Food Allergy Independent Measure, and productivity questions.

**Results:**

One hundred caregivers (55% female) of children with peanut allergy (aged 4–15 years) completed the survey. Male and female caregivers reported mean levels of anxiety significantly higher than United Kingdom population norms. Caregivers of children with severe peanut allergy reported significant impacts on their careers and health-related quality of life. Neither caregiver nor child gender impacted burden, indicating that male and female caregivers are equally anxious and suffer the same level of negative career, productivity, and health-related quality-of-life impact due to their child’s peanut allergy. Caregivers’ perceived risk of outcomes related to their child’s peanut allergy (e.g., death or severe reaction) as measured by the Food Allergy Independent Measure independently predicted burden.

**Conclusions:**

Caregivers of children with peanut allergy in the United Kingdom experience health-related quality-of-life, psychosocial, and productivity burden; this study demonstrates the high levels of anxiety reported by both male and female caregivers.

## Background

Peanut allergy affects between 0.6% and 1% of the adult population and between 1% and 3% of children in the developed world [1, 2], and studies suggest the prevalence is increasing in the United Kingdom (UK) [3]. There is currently no approved treatment for peanut allergy outside of the United States; therefore, management requires strict avoidance of peanuts and emergency treatment in the case of accidental ingestion.

Previous research has identified several areas of caregivers’ health-related quality of life (HRQL) that are affected by having a child with food allergy. Parents of children with food allergies report significantly greater emotional impact, impact on parental time, and limitations in usual family activities than do population norms in the United States [4]. Similarly, parents of children with food allergies report significantly greater emotional impact [5] and effect on social relationships [6] than do parents of children with no allergic diseases. An older study that used a convenience sample in comparison with rheumatological disease, a condition that is very dissimilar to allergy, found impairment of family relations reported by parents of children with peanut allergy [7]. In addition, parent-reported food allergy is associated with caregiver stress, although parents of children who have undergone an oral food challenge report significantly less stress [8]. Even with suspected food allergy prior to clinical diagnosis, the level of anxiety in parents was significantly higher than in those who had just been diagnosed with a chronic illness [9].

Many factors have been associated with increased caregiver burden for those with food allergies. Food allergy characteristics, including type and number of food allergens, type and severity of symptoms, previous anaphylactic reactions, adrenaline autoinjector use, and oral food challenge history have been found to influence parental burden [8, 10–14]. In addition, factors such as lower caregiver income and education, child’s age and age at most severe reaction, parental self-efficacy for food allergy management, and negativity experienced in social relationships have also been shown to influence caregiver burden [11, 12, 14, 15].

Limited research has explored whether caregiver gender influences the amount of burden experienced by caregivers of children with food allergies; some evidence suggests gender does make a difference. One study found that mothers reported significantly lower HRQL than fathers but greater empowerment, regardless of allergen severity, type of food allergy, or comorbidities [16]. Another study found that mothers reported significantly more negative impact than fathers regarding meal preparation, family social activities, stress, and free time, and significantly greater involvement in allergy-related care. However, fathers who reported more frequent medical appointment attendance perceived meal preparation as being significantly more impacted by food allergies than fathers who were less involved, suggesting that differences in impact may be related to the level of involvement in day-to-day activities rather than gender [17]. In peanut allergy specifically, a study of families including a child with peanut allergy found that mothers reported worse HRQL and more stress and anxiety than did fathers [18].

As outlined above, much previous research on caregiver impact has been conducted in food allergy in general, with limited studies focused on peanut allergy specifically. One study in peanut allergy found that a subset of parents of a child with peanut allergy experienced high levels of parenting stress [19]. Parents of a child with peanut allergy have also expressed difficulties with their child’s transition to independence and their own subsequent loss of control [20], suggesting that the child’s age may influence HRQL burden for caregivers. As discussed previously, one study found that caregiver gender may also influence caregiver burden in peanut allergy [18]. No research has previously explored the impact of peanut allergy specifically on caregiver work or productivity, although one survey of caregivers of children with any food allergy found that a small proportion (9%) reported a work-related impact of their child’s food allergy, such as career restrictions, job change, or job loss [21].

Thus, given the limited research on caregiver burden in peanut allergy specifically, the objectives of this study were to (1) describe the psychosocial and productivity impact of caring for a child with peanut allergy, (2) explore the effect of caregiver and child gender on caregiver HRQL, and (3) examine which factors predict caregiver HRQL in peanut allergy. To explore these objectives, we report on results from a survey of caregivers of children with peanut allergy aged 4 to 15 years in the UK.

## Methods

### Study design

This study used a cross-sectional online survey to explore burden in caregivers of children aged 4 to 15 years with peanut allergy in the UK. The study was reviewed and approved by the Freiburg Ethics Commission International (FECI) prior to participant recruitment (FECI code: 017/1938; date: 20 November 2017).

### Participants

Participants were recruited through a specialist survey recruitment panel. Participants were eligible for the study if they were a parent or primary caregiver of a child (aged 4–15 years) with a medically diagnosed peanut allergy, who had experienced at least one reaction to peanuts in their day-to-day life (not a food challenge). In order to ensure diversity in the severity of peanut allergy in the sample, a quota was set for a minimum of 80% of participants who perceived the severity of their child’s peanut allergy as moderate or severe, with a minimum of 25% in the moderate/severe population having needed to use an adrenaline autoinjector, and a minimum of 10% having experienced a life-threatening event.

### Procedure and measures

Eligible participants were provided with information about the study purpose and procedures. Participants were asked to read the information provided, and if interested in participating, were asked to give their consent online prior to completing the online survey. Participants received nominal reimbursement for participating in the study.

The survey consisted of sociodemographic questions about the caregiver and their child and clinical questions about the child’s peanut allergy. The clinical questions included the caregivers’ perception of the severity of their child’s peanut allergy (mild, moderate, or severe), caregivers’ confidence in managing their child’s reactions to peanut allergy (“not at all confident” to “very confident”), the number of reactions to peanut their child experienced in the last 12 months and in the child’s lifetime, the type of treatment received in the last 12 months and in their lifetime, and whether their child experienced a life-threatening reaction to peanut in the last 12 months and in their lifetime. Participants were also asked about the impact on their productivity due to their child’s peanut allergy, including the number of days they were absent from work in the last 12 months, the number of days their productivity was impacted in the past 12 months, and whether their career was impacted. The survey also included validated measures of caregiver HRQL (EQ-5D-5L, Food Allergy Quality of Life–Parental Burden [FAQL-PB]) and caregiver anxiety and depression (Hospital Anxiety and Depression Scale [HADS]) and a measure of caregiver food allergy-related expectation of outcome (Food Allergy Independent Measure-Parent Form [FAIM-PF]).

The EQ-5D-5L [22] is a generic, preference-based health status measure. Participants report their current health on five dimensions (mobility, self-care, pain and discomfort, usual activities, and anxiety and depression) with five response options for each dimension, from “no problems” to “extreme problems.” Responses are converted into a single index value that can be used in cost-effectiveness analyses, where a score of 1 represents “full health” and a score of 0 represents “dead.” The instrument was scored using the van Hout et al. [23] mapping algorithm, which maps to the UK value set [24]. Participants also rate their current health on a 0 to 100 visual analogue scale (VAS).

The FAQL-PB [25] was developed to assess burden for parents with food-allergic children. The FAQL-PB scale is a self-administered 17-item questionnaire. Each item is presented on a 7-point Likert scale ranging from 1 (not troubled) to 7 (extremely troubled). Items are summed to provide a total continuous score; higher scores indicate greater parental burden. The instrument can also be scored as two domains: limitations on life and emotional distress [26]. Validated for use in a UK sample, the scale has been found to have excellent reliability and validity with a Cronbach’s alpha of 0.952 for the overall scale, 0.952 for the limitations on life subscale, and 0.860 for the emotional distress subscale [26].

The HADS [27] is a 14-item measure of anxiety and depression used in both hospital and community settings. The questionnaire gives clinically meaningful results as a psychological screening tool and can help in assessing the symptom severity and clinical “caseness” of anxiety disorders and depression in patients with illness and in the general population. The HADS provides separate scores for anxiety and depression, ranging in each from 0 to 21, with 0–7 indicating “normal” or no anxiety or depression, 8–10 indicating mild, 11–14 indicating moderate, and 15–21 indicating severe anxiety or depression; scores of ≥ 11 indicate probable “caseness” [27]. Population norms related to the proportion of people reporting probable clinical caseness in the UK (based on adults aged 25 to 65 years) have been estimated at 12.5% (males) and 19% (females), with an average for males and females of 15.8% for anxiety and 6.9% for depression [28]. The HADS has been validated many times in different populations, with internal consistency for the anxiety scale ranging from α = 0.68–0.93 (mean: 0.83) and the depression scale ranging from α = 0.67–0.90 (mean: 0.82) [29].

The FAIM-PF [30] consists of four items related to the parent’s perception of their child’s likelihood of allergic reaction events (accidentally ingesting the food to which they are allergic, having a severe reaction, dying from their food allergy, effectively treating themselves when needed). Each item is answered on a 7-point scale, with FAIM scores ranging from 1 (extremely unlikely) to 7 (extremely likely).

### Analysis

Demographic and clinical characteristics were analysed using descriptive statistics. The FAQL-PB and HADS were scored according to their published scoring instructions [25–27]. In line with recommendations of the National Institute for Health and Care Excellence, EQ-5D-5L data were scored using the van Hout et al. [23] mapping function. Pearson’s, point biserial, and Spearman’s correlations (where data were not normally distributed) were conducted as appropriate to explore relationships between variables. Correlations were interpreted in line with Cohen’s guidelines: small: 0.10 to < 0.30; moderate: 0.30 to < 0.50; large: ≥ 0.50 [31]. To explore the impact of caregiver and child gender on caregiver burden, two-way ANOVA (analysis of variance) and Chi square tests were conducted. Chi square tests were also conducted to explore the association between peanut allergy severity and caregiver career impact. To further explore which outcomes significantly predicted caregiver impact, multivariate ordinary least squares regression models were conducted. To assess whether EQ-5D and HADS scores were significantly different from population norms, one-sample t-tests and one-sample tests of proportion were conducted as appropriate. Results of statistical tests were considered statistically significant if p values were below 0.05. All analyses were conducted using Stata version 16.0.

## Results

The survey information and link were sent to 1330 panel members; 100 caregivers were eligible and completed the survey. Demographics and clinical characteristics of the caregivers and their children are shown in Table 1. The caregivers had a mean age of 40 years (SD 7.6; range 21–65 years), and their children with peanut allergy had a mean age of 10 years (SD 3.4; range 4–15 years). The sample contained male (45%) and female (55%) caregivers. Parents and children had high proportions of allergic rhinitis (parents 22%, children 36%), asthma (parents 20%, children 34%), and skin disorders (parents 17%, children 26%).

**Table 1:** Caregiver and child demographic and clinical characteristics

Characteristic		Caregivers (N = 100)	Children (N = 100)
Age, years	Mean (SD)	39.49 (7.59)	9.82 (3.42)
	Min–max	21–65	4–15
Female	N	55	42
Ethnicity, n	White	85	86
	Black	4	0
	Asian	10	9
	Mixed race	1	5
Main occupation, n	Employed outside the home	89	–
	Looking after family and/or home	6	–
	Other	5	–
Comorbidities, n	Allergic rhinitis	22	36
	Asthma	20	34
	Eating disorder	7	6
	Skin disorder	17	26
	Stress	13	5
	Other	16	11
	None	37	28
Caregiver, food allergy, n	Peanut allergy	10	–
	Other food allergy	11	–
Child, food allergies other than peanut, n (%)	No	–	76 (76.0)
	Yes	–	24 (24.0)
If “Yes,” food allergy, n (%)	Celery	–	2 (8.3)
	Cow milk/dairy products	–	6 (25.0)
	Egg	–	11 (45.8)
	Fish/shellfish	–	1 (4.2)
	Soya beans/other legumes	–	1 (4.2)
	Other nuts	–	11 (45.8)
	Other	–	5 (21.0)
Prescribed an AAI, n	Yes		71
Experienced life-threatening event (lifetime n)	Yes		34
Number of reactions (lifetime)	Mean (SD)		11.98 (28.2)
Peanut allergy severity as reported by caregiver, n	Mild	–	13
	Moderate	–	66
	Severe	–	21

*AAI* adrenaline autoinjector, *n* sample size, *SD* standard deviation

### Impact of caregiving

**Fig. 1:**
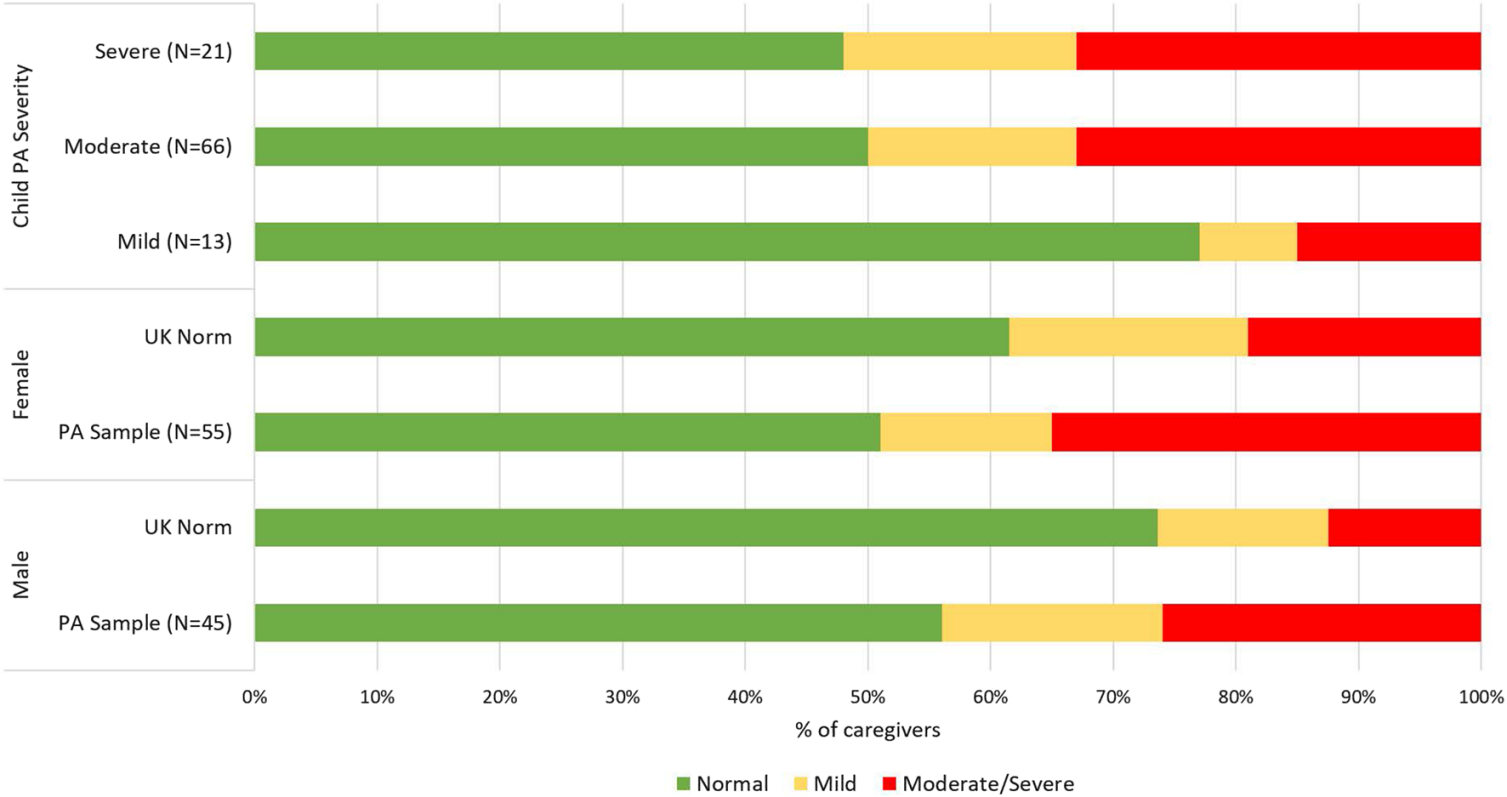
Proportion of HADS Anxiety categories (normal, mild, moderate/severe) by gender and child’s peanut allergy severity. *HADS* Hospital Anxiety and Depression Scale, *PA* peanut allergy, *UK* United Kingdom

Summaries of the impact of being a caregiver to a child with peanut allergy relative to levels of anxiety and depression as well as generic and food-allergy-specific measures of HRQL are presented in Table 2 and Fig. 1. All health-outcome measures were highly significantly correlated with each other (*r*’s = 0.42–0.65, p’s < 0.001), indicating medium to large effect sizes [31]. Table 2 shows that caregivers’ mean anxiety levels overall and for each subgroup (peanut allergy severity, caregiver gender) are all above the normal range (HADS normal range: scores of 0–7). The mean scores are significantly higher than the population norm [28] for the overall sample (t = 4.00, p < 0.001), males (t = 3.07, p < 0.001), females (t = 2.44, p < 0.05), and for those who rate their child’s peanut allergy as moderate (t = 3.44, p < 0.001) or severe (t = 2.73, p < 0.05). Caregivers’ depression mean scores for each subgroup all fall within the normal range (HADS score of 0–7) but are significantly higher than UK population norms overall (t = 2.26, p < 0.05). Similarly, caregiver rates of probable clinical anxiety (moderate/severe HADS scores of > 10) are approximately double the UK population norms (UK norms: mean = 15.75%, males = 12.5%, females = 19%) and significantly higher for the total sample (31%; z = 4.2, p < 0.001), males (26%, z = 2.9, p < 0.01), females (35%, z = 2.9, p < 0.001), and those reporting a child with moderate peanut allergy (33%, z = 3.9, p < 0.001) or severe peanut allergy (33%, z = 2.2, p < 0.05) (Fig. 1).

**Table 2:** Caregiver-reported measures of burden by gender and child peanut allergy severity

	N	EQ-5 D^a^		HADS^b^		FAQL-PB		
		Utility	VAS	Anxiety	Depression	Total score	Emotional distress	Life limitations
		Mean (SD)	Mean (SD)	Mean (SD)	Mean (SD)	Mean (SD)	Mean (SD)	Mean (SD)
Total sample	100	0.873 (0.209)	79.96 (17.70)*	8.09 (4.85)**	4.93 (4.20)*	2.98 (1.56)	2.96 (1.63)	3.02 (1.52)
Caregiver gender								
Male	45	0.867 (0.243)	79.00 (17.09)*	7.73 (4.86)**	4.89 (4.02)	3.00 (1.50)	2.98 (1.58)	3.03 (1.44)
Female	55	0.879 (0.179)	80.75 (18.30)	8.38 (4.87)*	4.96 (4.38)	2.97 (1.61)	2.95 (1.69)	3.01 (1.59)
Child PA severity								
Mild	13	0.913 (0.204)	83.08 (11.43)	5.92 (4.23)	4.31 (3.99)	2.48 (1.37)	2.50 (1.48)	2.45 (1.21)
Moderate	66	0.888 (0.185)	80.18 (17.41)*	8.26 (4.98)**	5.23 (4.44)*	2.93 (1.58)	2.92 (1.65)	2.95 (1.54)
Severe	21	0.803 (0.273)	77.33 (21.77)	8.91 (4.63)*	4.38 (3.57)	3.45 (1.54)	3.40 (1.64)	3.56 (1.49)

*FAQL*-*PB* Food Allergy Quality of Life-Parental Burden, *HADS* Hospital Anxiety and Depression Scale, *PA* peanut allergy, *SD* standard deviation, *UK* United Kingdom, *VAS,* visual analogue scale

^a^ EQ-5D: Utility index scores range from 0 (dead) to 1 (full health); VAS ranges from 0 to 100. UK population norm: 18–64-year-olds: overall: utility: 0.885, VAS: 84.72; males: mean utility = 0.880, mean VAS = 85.22; females: mean utility = 0.888, mean VAS = 84.38 [32]

^b^ Scores range from 0 to 21: 0–7 = “normal” or no anxiety or depression, 8–10 = mild, 11–14 = moderate, 15–21 = severe anxiety or depression. Scores of ≥ 11 indicate probable “caseness.” UK population norms: anxiety = 6.15, depression = 3.98 [28]

*Significantly different from UK population norms (p < 0.05)

**Significantly different from UK population norms (p < 0.001)

Overall, generic HRQL, as measured by the EQ-5D utility index, was similar to UK population norms, although VAS scores overall were significantly lower than population norms (UK norm for those aged 18–64 years mean utility overall = 0.885, mean VAS = 84.72 [32]; VAS compared to population norm: t = − 2.69, p < 0.05). However, in the subgroup that perceived their children to have severe peanut allergy, generic HRQL was lower than norms using both utility index (mean 0.803, population norm 0.885) and VAS (mean 77.22, population norm 84.72), yet these differences were not significant (p > 0.05) considering the small sample size of the “severe” subgroup. Disutility associated with having a child with increased peanut allergy severity ranged from 0.025 (mild utility = 0.913 vs moderate utility = 0.888) to 0.11 (mild utility = 0.913 vs severe utility = 0.803), with a mean disutility of 0.068 (see Table 2 for mean utility values). The utility decrement was largely driven by the EQ-5D anxiety and depression domain: 44% of caregivers reported being at least slightly anxious or depressed, compared with those reporting at least slight problems with self-care (7%), mobility (9%), usual activities (17%), and pain or discomfort (19%).

**Fig. 2:**
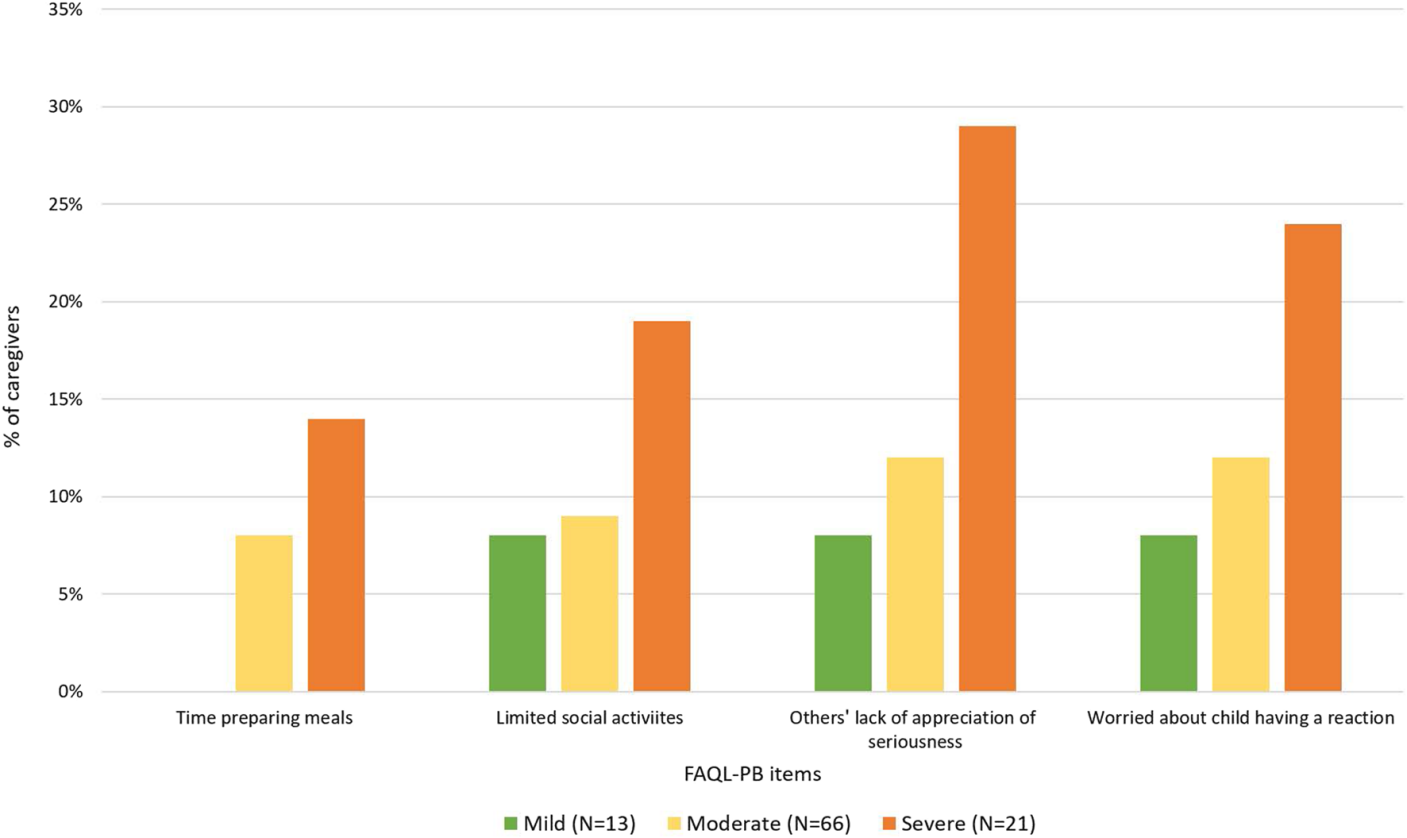
Caregivers who reported being very/extremely impacted on FAQL-PB items, by child’s peanut allergy severity group. *FAQL*-*PB* Food Allergy Quality of Life–Parental Burden

The FAQL-PB food-allergy-specific quality-of-life data suggest minimal (mean scores of 2–3) to moderate (mean scores of 3–4) levels of trouble/burden. The results show a statistically significant increase in caregiver burden among those with children reported to have severe versus mild peanut allergy in the FAQL-PB limitations on life domain (B = 6.64, 95% CI 0.36–12.92, p < 0.05). To illustrate this burden with more concrete descriptive examples of the impact, Fig. 2 shows the proportion of caregivers who reported being “very” or “extremely” burdened with respect to specific activities and concerns (preparing meals, social limitations, others’ attitudes, fear of reactions) according to their child’s peanut allergy severity.

Table 3 presents absenteeism, productivity, and career impact results. The overall mean number of days absent in the last 12 months and productivity impacted among caregivers currently in paid employment due to their child’s allergy were M = 1.6 (SD: 2.2) and M = 1.9 (SD: 3.6), respectively. Absenteeism and productivity impacts increased with severity of child’s allergy, but not significantly (p > 0.05); however, career impact was significantly associated with increased severity (χ^2^ = 9.19, p = 0.01), with 43% of caregivers of children with severe peanut allergy reporting some career impact (e.g., having to change job or stop work or having restricted career choices).

**Table 3:** Caregiver-reported absenteeism, productivity, and career impact by gender and child peanut allergy severity

	Currently employed			Total Sample	
	N	Days absent from work (last 12 months)	Days productivity impacted (last 12 months)	N	Negative career impact (overall): Yes
		Mean (SD)	Mean (SD)		%
Total	91	1.60 (2.20)	1.87 (3.64)	100	22
Caregivers by gender					
Male	45	1.87 (2.36)	1.71 (2.73)	45	23
Female	46	1.35 (2.01)	2.02 (4.38)	55	22
Severity of child’s PA					
Mild	12	1.08 (2.47)	0.50 (1.24)	13	0
Moderate	61	1.67 (2.12)	1.84 (3.84)	66	20
Severe	18	1.72 (2.35)	2.89 (3.83)	21	43

*N* sample size, *PA* peanut allergy

#### Relationship between gender and caregivers’ burden

Neither caregiver gender nor child gender were significantly correlated with any of the measures of caregiver burden (all *r*’s < 0.2, all p’s > 0.05). ANOVAs and Chi square tests showed that caregiver and child gender did not have any significant main or interaction effects on any of the measures of caregiver burden (all p’s > 0.05).

### Factors impacting caregiver burden

The significant correlations between measures of burden and potential predictors of burden are detailed in Table 4. All other potential predictors (adrenaline autoinjector use, caregiver and child gender, child age) were not significantly associated with outcome assessments.

**Table 4:** Correlations between caregiver burden outcomes and potential predictors of burden

	EQ-5D		FAQL-PB			HADS	
	Utility	VAS	Total	Emotional	Limitations	Anxiety	Depression
Proxy-reported severity	− 0.201*	− 0.035	0.187	0.167	0.208*	0.168	0.004
Confidence	0.004	0.199*	− 0.229*	− 0.203*	− 0.266**	− 0.215*	− 0.323**
Life threat, past 12 mo	− 0.330***	− 0.248*	0.324**	0.327***	0.295**	0.250*	0.156
Life threat, lifetime	− 0.278**	− 0.213*	0.302**	0.286**	0.312**	0.152	0.014
Reactions, past 12 mo	− 0.262**	− 0.354***	0.385***	0.383***	0.380***	0.226*	0.344***
Reactions, lifetime	− 0.317**	− 0.327***	0.377***	0.367***	0.379***	0.239*	0.235*
FAIM Total	− 0.398***	− 0.344***	0.602***	0.588***	0.587***	0.400***	0.379***
FAIM Ingest peanut	− 0.349***	− 0.387***	0.617***	0.612***	0.584***	0.440***	0.444***
FAIM Severe reaction	− 0.263**	− 0.220*	0.438***	0.423***	0.440***	0.336**	0.258**
FAIM Dying	− 0.418***	− 0.360***	0.551***	0.537***	0.542***	0.398***	0.410***
FAIM Treatment	0.268**	− 0.161	0.368***	0.360***	0.358***	0.240*	0.131

*FAIM* Food Allergy Independent Measure, *FAQL*-*PB* Food Allergy Quality of Life–Parental Burden, *HADS* Hospital Anxiety and Depression Scale, *VAS* visual analogue scale

* p < 0.05, ** p < 0.01, *** p < 0.001

Multivariate regression models for each outcome assessment are presented in Table 5. As the number of reactions in the past 12 months and in the child’s lifetime are not independent of each other, these were run in separate models. Also, given the high correlations between FAIM items and total FAIM score, only the total score was included in the models. The regression models show that for all measures of caregiver burden, the caregivers’ increased expectation of negative outcomes as measured by the FAIM (e.g., perception of risk of dying, having a severe reaction, or accidental ingestion of allergen) significantly increased burden. For all except EQ-5D utility, caregiver confidence also significantly predicted burden, with higher confidence associated with lower burden. The number of reactions in the past 12 months was the only other relatively consistent predictor of burden (EQ-5D VAS, FAQL-PB total and limitation domain, and HADS depression), with significantly increased burden associated with 2 or more reactions in the past 12 months. All models were repeated with “lifetime” reactions or life-threatening events, but these were only significant predictors for (1) FAQL-PB limitations domain, where having had a life-threatening event was associated with a 3-point increase in score (B = 2.97, p < 0.05) and (2) EQ-5D VAS, where having had ≥ 6 reactions in one’s lifetime versus zero reactions was associated with a 9-point decrease on the VAS (B = − 8.90, p < 0.05). The significant association between a child’s proxy-reported severity and FAQL-PB total score, and between severity and EQ-5D utility, were no longer significant when other predictors were considered.

**Table 5:** Multiple regression models with caregiver burden outcome measures as dependent variables

Outcome/predictors		B	SE	95% CI	
				Lower	Upper
EQ-5D Utility					
FAIM Total		− 0.011**	0.004	− 0.020	− 0.003
Life-threat. in past 12 mo (Ref: No)	Yes	− 0.089	0.046	− 0.181	0.004
Reactions in past 12 mo, n (Ref: None)	1	− 0.015	0.060	− 0.133	0.104
	2 or more	− 0.076	0.054	− 0.183	0.032
Proxy-reported severity (Ref: Mild)	Moderate	− 0.017	0.058	− 0.132	0.097
	Severe	− 0.053	0.069	− 0.190	0.084
EQ-5D VAS					
FAIM total		− 0.907**	0.341	− 1.583	− 0.230
Confidence		3.978*	1.947	− 0.128	7.568
Life-threat. in past 12 mo (Ref: No)	Yes	− 4.135	3.937	− 11.952	3.682
Reactions in past 12 mo, n (Ref: None)	1	− 3.706	4.984	− 13.602	6.190
	2 +	− 9.394*	4.585	− 18.497	− 0.290
HADS anxiety					
FAIM total		0.336**	0.927	0.149	0.518
Caregiver confidence		− 1.287*	0.530	− 2.338	− 0.235
Life-threat. in past 12 mo (Ref: No)	Yes	1.377	1.071	− 0.750	3.504
Reactions in past 12 mo, n (Ref: None)	1	0.822	1.356	− 1.871	3.515
	2+	1.044	1.248	− 1.433	3.521
HADS depression					
FAIM_Q1_sum		0.272**	0.076	0.121	0.424
Caregiver confidence		− 1.557**	0.436	− 2.423	− 0.691
Life-threat. in past 12 mo (Ref: No)	Yes	− 0.134	0.882	− 1.886	1.617
Reactions in past 12 mo, n (Ref: None)	1	1.988	1.117	− 0.229	4.206
	2 +	2.750**	1.027	0.710	4.790
FAQL-PB total					
FAIM total		2.766***	0.420	1.931	3.601
Caregiver confidence		− 7.321**	2.402	− 12.091	− 2.553
Life-threat. in past 12 mo (Ref: No)	Yes	8.871	6.150	− 3.340	21.081
Reactions in past 12 mo, n (Ref: None)	1	8.871	6.150	− 3.340	21.081
	2 +	12.228*	5.657	− 0.995	23.461
FAQL-PB life limitations					
FAIM total		0.897***	0.150	0.599	1.196
Caregiver confidence		− 2.959**	0.830	− 4.606	− 1.311
Life-threat. in past 12 mo (Ref: No)	Yes	1.710	1.663	− 1.593	5.013
Reactions in past 12 mo, n (Ref: None)	1	4.176	2.134	− 0.061	8.414
	2 +	4.748*	1.946	0.883	8.612
Proxy-reported severity (Ref: Mild)	Moderate	2.789	2.063	− 1.308	6.886
	Severe	4.157	2.468	− 0.745	9.059
FAQL-PB emotions					
FAIM Total		1.828***	0.294	1.245	2.412
Caregiver confidence		− 4.427*	1.678	− 7.759	− 1.095
Life-threat. in past 12 mo (Ref: No)	Yes	5.067	3.394	− 1.672	11.806
Reactions in past 12 mo, n (Ref: None)	1	5.239	4.296	− 3.292	13.769
	2 +	7.672	3.952	− 0.175	15.520

*B* standardized beta coefficient, *CI* confidence interval, *FAIM* Food Allergy Independent Measure, *FAQL*-*PB* Food Allergy Quality of Life – Parental Burden, *HADS* Hospital Anxiety and Depression Scale, *Ref* reference category, *SE* standard error, *VAS* visual analogue scale

* p < 0.05, ** p < 0.01, *** p < 0.001

## Discussion

This study helps provide a better understanding of the impact, and its driving factors, associated with being a caregiver to a child with peanut allergy in the UK. The study highlighted the psychological burden experienced by caregivers of children with peanut allergy, revealing levels of anxiety and depression significantly higher than UK population norms for males (anxiety and depression) and females (anxiety only) and rates of probable clinical anxiety significantly higher than the UK population. There was a trend toward poorer health utility values among caregivers who perceived their child to have severe peanut allergy relative to the general population, although differences were not statistically significant, likely due to the small sample size of the “severe” group. These peanut-allergy-specific caregiver utility values are the first to be published and indicate that the HRQL burden for caregivers of children with peanut allergy is largely driven by the levels of anxiety and depression experienced. Furthermore, caregivers of children with severe peanut allergy also reported significant impacts on their careers and limitations to their HRQL. Notably, caregiver gender did not impact burden, as the study indicated that male and female caregivers are equally anxious and suffer the same level of negative career, productivity, and HRQL impact due to their child’s peanut allergy.

This study found that caregivers’ expectations of outcome, specifically their perception of their child’s risk of allergen exposure, having a severe reaction, dying, or being able to effectively treat themselves (measured using the FAIM) independently predicted all measures of caregiver burden. Caregiver confidence was also an independent predictor of burden (for all measures except utility). Objective measures of severity such as life-threatening events and number of reactions were also significantly associated with burden; however, these did not retain significance in multivariate models, suggesting that these events are captured within the measures of caregivers’ expectations and levels of confidence.

The results of this study support previous research showing a negative impact of peanut allergy on caregiver HRQL [18]. In contrast to previous research showing a gender difference in caregiver burden in peanut allergy and food allergy, the current study suggests there is no difference, as both male and female caregivers reported a negative impact. Similarly, comparable levels of career impact were reported by male and female caregivers, suggesting equal involvement in peanut-allergy-related care. This may support previous findings that any gender differences may be related to the level of involvement rather than gender [17]. In addition, previous research showing differences in responses based on the gender of caregivers of children with peanut allergy [18] used a smaller sample size recruited from a specialist allergy center and was published 10 years ago; the more current data reported here may reflect increased shared responsibility between parents.

This study found that caregiver expectation of outcome measured using FAIM was the key driver of caregiver burden in peanut allergy; this is in contrast to previous research that found the FAIM was not a significant predictor of caregiver psychosocial impact [33]. However, caregiver self-efficacy (confidence) has been shown to predict the psychosocial impact of food allergy [14], which is consistent with the current study’s finding that caregiver confidence predicted several measures of caregiver burden. A number of studies have shown that food allergy reaction history impacts caregiver burden [8, 10, 11], although these studies did not include FAIM in the same regression model. In the current study, reaction history in terms of the number of reactions and having experienced a life-threatening event showed some impact on caregiver burden but was not the main driver when parental expectation of outcome (measured by FAIM) was also included.

Some limitations should be considered when interpreting the results of this study. The research was conducted in the UK only; therefore, the results may not be generalizable to caregivers in other countries. In addition, the sample size of 100 is relatively small and so some of the analyses may be underpowered, such as the differences between the severe peanut allergy population versus general population norms. Another limitation is that the severity of peanut allergy can be difficult to objectively measure, given that food allergic reactions are unpredictable, and severity is not determined by test results or history of reaction; clinical severity as reported by parents should be considered a subjective measure. Thus, it is important to interpret results of analyses exploring associations between peanut allergy severity and caregiver impact as reflecting caregiver perception of severity, rather than clinical severity itself. Future research could expand on this study by increasing the sample size, exploring parent and child dyads, and exploring the burden experienced by caregivers of children with peanut allergy in other countries.

## Conclusions

This study highlights the burden experienced by caregivers of children with peanut allergy in the UK, particularly the high levels of anxiety reported by both male and female caregivers. In addition, caregivers of children with severe peanut allergy report significant impacts on their careers and limitations in their HRQL. The study also found that caregiver expectation of outcome, confidence, and several objective measures of severity were significantly associated with caregiver burden.

## Data Availability

Data are available upon reasonable request to the corresponding author.

## Abbreviations

FAIM-PF: Food Allergy Independent Measure–Parent Form
FAQL-PB: Food Allergy Quality of Life–Parental Burden
HADS: Hospital Anxiety and Depression Scale
HRQL: Health-related quality of life
VAS: Visual analogue scale
